# MicroRNA miR-627-5p restrains pulmonary artery smooth muscle cell dysfunction by targeting MAP 2 K4 and PI3K/AKT signaling

**DOI:** 10.1186/s41021-022-00251-4

**Published:** 2022-09-27

**Authors:** Ting Li, Xiaoqin Tan, Yuexia Huang, Jun Cui, Fan Chen, Ying Xiong

**Affiliations:** 1 Department of Respiratory and Critical Care Medicine, Wuhan Fourth Hospital, Wuhan, Hubei 430000 China; 2 Wuhan Fourth Hospital, No. 473, Hanzheng Street, Qiaokou District, Wuhan, Hubei China

**Keywords:** Chronic obstructive pulmonary disease, Pulmonary artery smooth muscle cell, miR-627-5p, MAP2K4, The PI3K/AKT pathway

## Abstract

**Background:**

Chronic obstructive pulmonary disease (COPD) is characterized by pulmonary vascular remodeling, which can be caused by abnormal proliferation and migration of pulmonary artery smooth muscle cells (PASMCs). Several microRNAs were demonstrated to regulate the PASMC dysfunction. Our study intends to evaluate whether miR-627-5p affects cigarette smoke extract (CSE)-induced aberrant biological behaviors of PASMCs.

**Methods:**

PASMCs was treated with CSE to create the in vitro cellular model of COPD. The viability and LDH release of PASMCs was detected by CCK-8 assay and LDH release assay. MiR-627-5p and MAP 2 K4 expression in CSE (2%)-treated PASMCs was detected by qRT-PCR. PASMC proliferation was observed under a microscope, and PASMC migration was assessed by Transwell migration assays. The binding of miR-627-5p on MAP 2 K4 was verified by dual-luciferase reporter assay. Protein levels of MAP2K4 and the PI3K/AKT signaling markers were examined by western blotting.

**Results:**

The viability of PASMCs treated with 2% CSE reached a peak. CSE dose-dependently downregulated miR-627-5p expression in PASMCs. MiR-627-5p overexpression attenuated the CSE-induced abnormal proliferation and migration of PASMCs. However, MAP2K4 overexpression antagonized the effects of miR-627-5p on PASMC dysfunction. Importantly, miR-627-5p inhibited CSE-stimulated activation of the PI3K/AKT pathway via downregulating MAP2K4.

**Conclusion:**

MiR-627-5p improves CSE-induced abnormal proliferation and migration of PASMCs by inhibiting MAP2K4 expression and the PI3K/AKT pathway.

## Introduction

Chronic obstructive pulmonary disease (COPD) ranks the third among leading causes of mortality worldwide, with a rising prevalence over the past several decades [1, 2]. Pulmonary vascular remodeling, which is regarded as a crucial pathological feature during the pathogenesis of COPD, contributes to pulmonary arterial hypertension (PAH) [3, 4]. Pulmonary vascular remodeling is primarily caused by dysregulated proliferation and migration of pulmonary artery smooth muscle cells (PASMCs) [5, 6]. It is estimated that at least 75% of COPD deaths are caused by cigarette smoke (CS), known as the principal risk factor for COPD [7]. CS can stimulate the production of mediators such as endothelin and endothelial nitric oxide synthase, which might induce abnormal proliferation of PASMCs [8, 9]. Thus, the molecular mechanisms underlying PASMC dysfunction including the aberrant proliferation and migration need to be explored for improving PAH and thereby alleviate the development of COPD.

MicroRNAs (miRNAs) are single-stranded non-coding RNA molecules of about 21–23 nucleotides in length [10]. MiRNAs usually inhibit gene expression in post-transcriptional level by binding with the 3′ untranslated region (UTR) of specific mRNA targets to induce mRNA degradation or repress translation [11]. MiRNAs influence multiple cellular processes, including growth, apoptosis, differentiation, and migration, and play a pivotal role in many human diseases [12]. Several miRNAs were reported to modulate the growth and migration of PASMCs, thereby mediating the progression of COPD. For example, miR-214–3p knockdown represses proliferation and migration, while and aggravates apoptosis of hypoxia-treated PASMCs by targeting rho guanine nucleotide exchange factor 12 (ARHGEF12) [13]. MiR-339 suppresses FGF2-induced proliferation of PASMCs via downregulating fibroblast growth factor receptor substrate 2 (FRS2) [14]. The upregulated miR-15a-5p in rats with PAH targets the vascular endothelial growth factor (VEGF)/p38/MMP-2 pathway, thereby attenuating PASMC proliferation and inducing apoptosis [15]. Previously, miR-627 was discovered to be downregulated in pulmonary artery homogenates isolated from lung tissues of COPD patients compared to non-smokers [16]. It was reported in pulmonary fibrosis, a kind of lung disorder, that miR-627 overexpression was reported to alleviate the abnormal proliferation of fibroblasts [17]. However, whether miR-627-5p affects the growth and migration of human PASMCs and participates in COPD development is unknown. In addition, by using bioinformatic tools, mitogen-activated protein kinase kinase 4 (MAP2K4) was discovered as a target of miR-627-5p. MAP2K4 was previously discovered to be upregulated in adipose tissues of patients with very severe COPD [18]. Therefore, we assumed that miR-627-5p is implicated in COPD development by targeting MAP2K4.

This study aimed to figure out the role of the miR-627-5p/MAP2K4 axis during COPD development. We proposed an assumption that miR-627-5p regulates the CS extract (CSE)-induced abnormal proliferation and migration of human PASMCs by targeting MAP2K4. The findings might provide a novel target for improving COPD development.

## Materials and methods

### Cell culture

Human PASMCs were bought from Sciencell (San Diego, CA, USA) and were incubated in smooth muscle cell medium (Sciencell) in a humidified atmosphere of 5% at 37 °C. PASMCs were passaged by trypsinization using 0.05% trypsin/EDTA (Invitrogen), and passages 3–5 cells were used for further experiments.

### CSE preparation

CSE was prepared as previously described [19]. Briefly, the smoke of a research cigarette (2R4F; Tobacco Health Research, University of Kentucky, KY, USA) was generated by a respiratory pump (Apparatus Rodent Respirator 680; Harvard, Germany) through a puffing mechanism related to the human smoking pattern (3 puffs·min^− 1^; 1 puff 35 mL; each puff of 2 s duration with 0.5 cm above the filter) and was bubbled into a flask containing 25 mL of DMEM. Then, the CSE solution was passed through a 0.22 μm filter for sterilization and was regarded as “100%” strength. CSE solution was standardized by measuring the absorbance at a wavelength of 320 nm, which is the specific absorption wavelength of peroxynitrite. In this system, a 100% CSE stock solution corresponds to 1 cigarette/25 mL medium. As the average blood volume of a person is approximately 5 L, a dilution to 1, 2, 5, and 10% CSE is equivalent to the average intake of a smoker who smokes 4, 8, 20, and 40 cigarettes per day. To evaluate the influence of CSE on PASMC proliferation, PASMCs were treated with 1, 2, 5 and 10% CSE, and the cell viability was evaluated by cell counting kit-8 (CCK-8) assay and lactate dehydrogenase (LDH) release assay.

### Cell transfection

MiR-627-5p mimics (for overexpressing miR-627-5p) and NC mimics (negative control), which were bought from GenePharma (Shanghai, China), were transfected into PASMCs using Lipofectamine 3000 (Invitrogen). For overexpression of MAP2K4, the full-length sequences of MAP2K4 were amplified and subcloned into pcDNA3.1 (Sangon, Shanghai, China) plasmid to produce pcDNA3.1/MAP2K4, which was also transfected into PASMCs using Lipofectamine 3000. Cells were harvested 48 h after transfection and subjected to qRT-PCR to evaluate the transfection efficiency.

### Quantitative real-time polymerase chain reaction (qRT-PCR)

Total RNAs were extracted from PASMCs using TRIzol reagent (Invitrogen). Then, 1 μg of total RNA was synthesized into complementary DNA (cDNA) using the RevertAid First Strand cDNA Synthesis Kit (Thermo Fisher Scientific, Waltham, MA, USA). qRT-PCR was performed using the SensiFAST SYBR No-ROX kit (Bioline, Taunton, USA) on a CFX RTPCR system (Bio-Rad，Hercules，MA，USA). The relative gene expression was quantified using the 2^−ΔΔCt^ method and normalized against the control genes, U6 small nuclear RNA for miR-627-5p and β-actin for mRNAs.

### CCK-8 assay

The viability of PASMCs after different treatment was assessed using CCK-8 (Dojindo, Kumamoto, Japan). PASMCs were seeded into the 96-well plates (4× 10^4^ cells/well). Next, 10 μL of CCK-8 solution was added into each well, followed by incubation at 37 °C for 2 h. The absorbance at 450 nm was determined using a microplate reader (Invitrogen).

### LDH release assay

An LDH Cytotoxicity Assay Kit (Roche Diagnostics, Indianapolis, USA) was used to examine the release of LDH. In brief, cells after different treatment or transfection were incubated in 96-well plates. At 1 h prior to detection, LDH release reagent was added. Then, 60 μL LDH detection working fluid was added into each well, followed by incubation at room temperature in the dark for 30 min. The absorbance at 490 nm was measured by using a microplate reader (Bio-Rad, Hercules, CA, USA). LDH activity was presented as the fold of the control group.

### Cell counting under a light microscopy

PASMCs were plated in 24-well plates (7× 10^3^cells/well) and incubated overnight. Then, cells were cultured in serum-free DMEM for 24 h, followed by different treatments. Finally, the images were captured using a light microscopy. Cells were harvested and a hemocytometer was applied for cell counting.

### Transwell migration assay

Transwell chambers (8 μm pore size; Corning) was employed for the assessment of the migratory potential of PASMCs after different treatment or transfection. Briefly, 100 μL cell suspension containing 5 × 10^4^ cells were seeded into the upper chamber with serum-free medium, The Transwell chamber was placed into 24-well plates filled with 500 μL complete medium (DMEM + 10% FBS). After incubation for 24 h, the non-migratory cells were removed with a cotton-tipped swab. The migrated cells were fixed with 4% formaldehyde solution for 10 min, and stained with 0.4% crystal violet for 15 min. The migrated PASMCs were observed and counted using a light microscope.

### Dual-luciferase reporter assay

Dual-luciferase reporter assay was conducted to validate the binding site of miR-627-5p on MAP2K4 3′-UTR predicted at TargetScan website (http://www.targetscan.org/). The 3’UTR of MAP2K4 was amplified from human genomic DNA and cloned into pmiR-RB-REPORT vector (RiboBio, Guangzhou, China). For the construction of mutational 3’UTR report vector, the region that base-paired with miR-627-5p seeding sequences were mutated through site-directed mutagenesis. Then, pmiR-RB-REPORT-MAP2K4–3′UTR-Wt (or Mut) was transfected into miR-627-5p overexpressing PASMCs and control cells using Lipofectamine 3000. A Dual-Luciferase Assay kit (Promega, Madison, WI) was used to detect the renilla and firefly luciferase activities 48 h post-transfection.

### Western blotting

Total proteins were isolated from PASMCs with RIPA reagents (Abcam, Cambridge, MA) and quantified by BCA kit (Beyotime, Shanghai, China). Then, 20 μg protein was separated by 10% SDS-PAGE and transferred onto PVDF membranes. The membranes were blocked for 1 h with 5% nonfat milk, followed by incubation with primary antibodies (Abcam) including anti-MAP2K4 (ab186125; 1:2000), anti-PI3K (ab180967; 1:2000), anti-AKT (ab179463; 1:10000), anti-p-PI3K (ab182651; 1:1000), anti-p-AKT (ab38449; 1:1000), anti-c-JUN (ab31419; 1000) and anti-β-actin (ab252556; 1:1000) overnight at 4 °C and subsequently the corresponding HRP-conjugated secondary antibody (Abcam) for 1 h at room temperature. Protein bands were visualized with an enhanced chemiluminescence kit (Pierce, Rockford, IL) and quantified using ImageJ software (NIH, Bethesda, MD).

### Statistical analysis

Data from at least three independent experiments were analyzed using the GraphPad Prism 6 (GraphPad Software, La Jolla, CA, USA), and are expressed as the mean ± SD. Student’s *t*-test or one-way ANOVA followed by Dunnett post hoc test was used to compare two groups or multiple groups. Differences were considered to have statistical significance when *p* < 0.05.

## Results

### CSE influences the viability of PASMCs in a dose-dependent manner

**Fig. 1:**
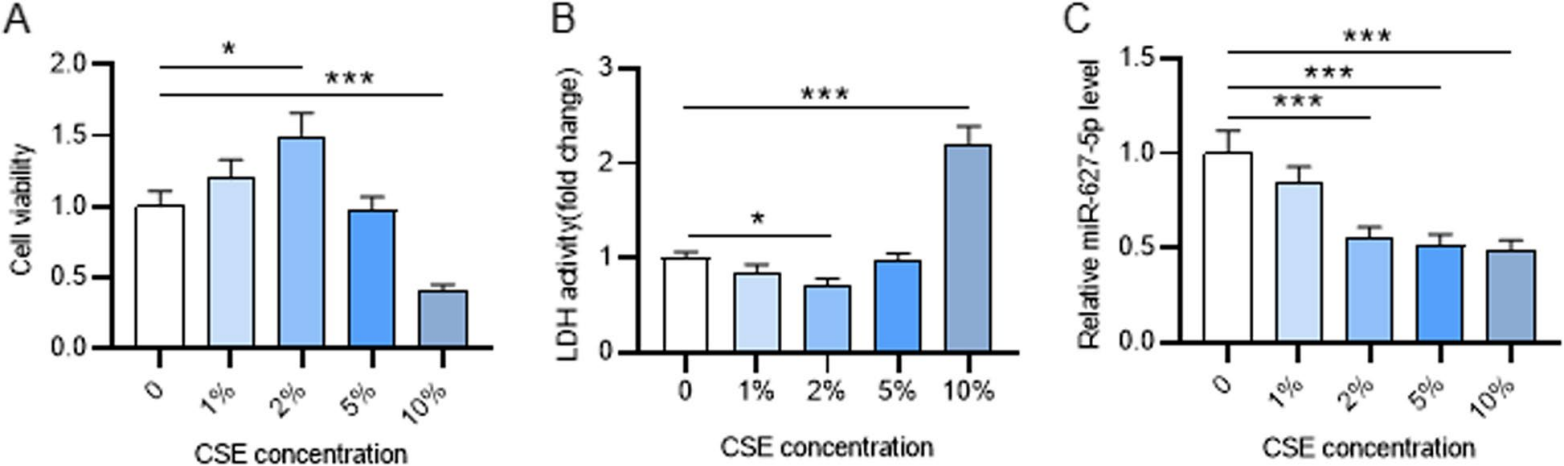
Influence of CSE on the proliferation of PASMCs. **A** CCK-8 assay of PASMCs viability following treatment with different concentrations of CSE. **B** The measurement of the lactate dehydrogenase (LDH) release of PASMCs by using an LDH Cytotoxicity Assay Kit. **C** qRT-PCR analysis of miR-627-5p level in PASMCs after treatment with different concentrations of CSE. *p<0.05, ***p<0.001

First, PASMCs were treated with different concentrations of CSE and subjected to CCK-8 assay to evaluate cell viability. The results demonstrated that versus the 0% CSE group, cell viability was enhanced in the 1 and 2% group. In the 5% CSE group, cell viability showed no significant change, while in the 10% CSE group, cell viability was significantly attenuated (Fig. 1A). LDH release assay revealed that LDH activity in the cell culture supernatant was attenuated after 1 and 2% CSE treatment, but was markedly enhanced after 10% CSE treatment (Fig. 1B). Since the peak cell viability was discovered after 2% CSE treatment, the following experiments were conducted using 2% CSE-treated PASMCs. Additionally, miR-627-5p expression in PASMCs treated with different concentrations of CSE was evaluated. PCR analysis indicated that CSE at concentrations of 2, 5 and 10% led to a remarkable decrease in miR-627-5p level (Fig. 1C). This indicated that miR-627-5p might regulate the biological functions of PASMCs.

### MiR-627-5p represses CSE-treated PASMC proliferation and migration

**Fig. 2:**
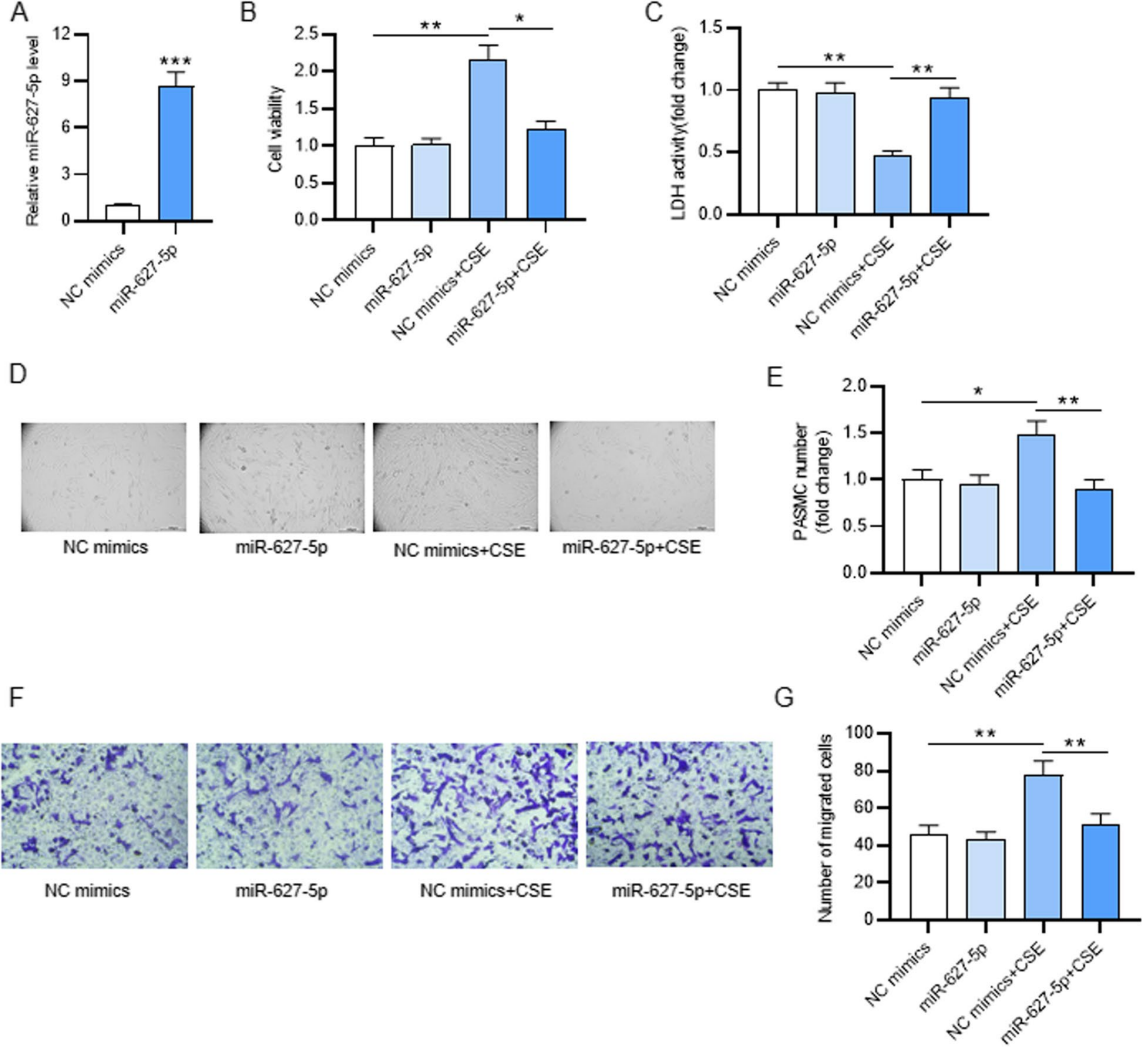
The influence of miR-627-5p on proliferation and migration of CSE-treated PASMCs. **A** qRT-PCR analysis of miR-627-5p level in PASMCs after overexpressing miR-627-5p. **B** CCK-8 assay of CSE-treated PASMC viability after miR-627-5p overexpression. **C** The detection of the lactate dehydrogenase (LDH) release of CSE-treated PASMCs after overexpressing miR-627-5p by using an LDH Cytotoxicity Assay Kit. **D** The images of PASMCs after different treatment under a light microscopy. **E** PASMC number in different groups were counted with a hemocytometer. **F-G** Transwell assay of PASMCs migration in each group. *p<0.05, **p<0.01, ***p<0.001

As shown by qRT-PCR, miR-627-5p level in PASMCs was significantly elevated after miR-627-5p overexpression (Fig. 2A). PASMCs were first treated with 2% CSE and then transfected with miR-627-5p mimics. CCK-8 assay indicated that the viability of PASMCs was enhanced after CSE treatment, which was reversed after overexpressing miR-627-5p (Fig. 2B). The reduction in LDH release by CSE-treated PASMCs was antagonized after overexpressing miR-627-5p (Fig. 2C). Then, PASMCs in each group were observed under a microscope. We discovered that PASMC number was increased after CSE treatment, while overexpressing miR-627-5p reversed the increase in PASMC number induced by CSE treatment (Fig. 2D-E), indicating that miR-627-5p overexpression inhibits CSE-induced abnormal proliferation of PASMCs. In addition, the migration of CSE-treated PASMC after miR-627-5p overexpression was also detected through Transwell migration assay. The NC mimics + CSE group displayed more migrated cell number than the NC mimics group, revealing that the migration of PASMCs was facilitated by CSE. However, overexpressing miR-627-5p reduced the number of migrated CSE-treated PASMCs (Fig. 2F-G). Therefore, miR-627-5p suppresses the CSE-induced migration of PASMCs.

### MAP2K4 directly targets miR-627-5p

**Fig. 3:**
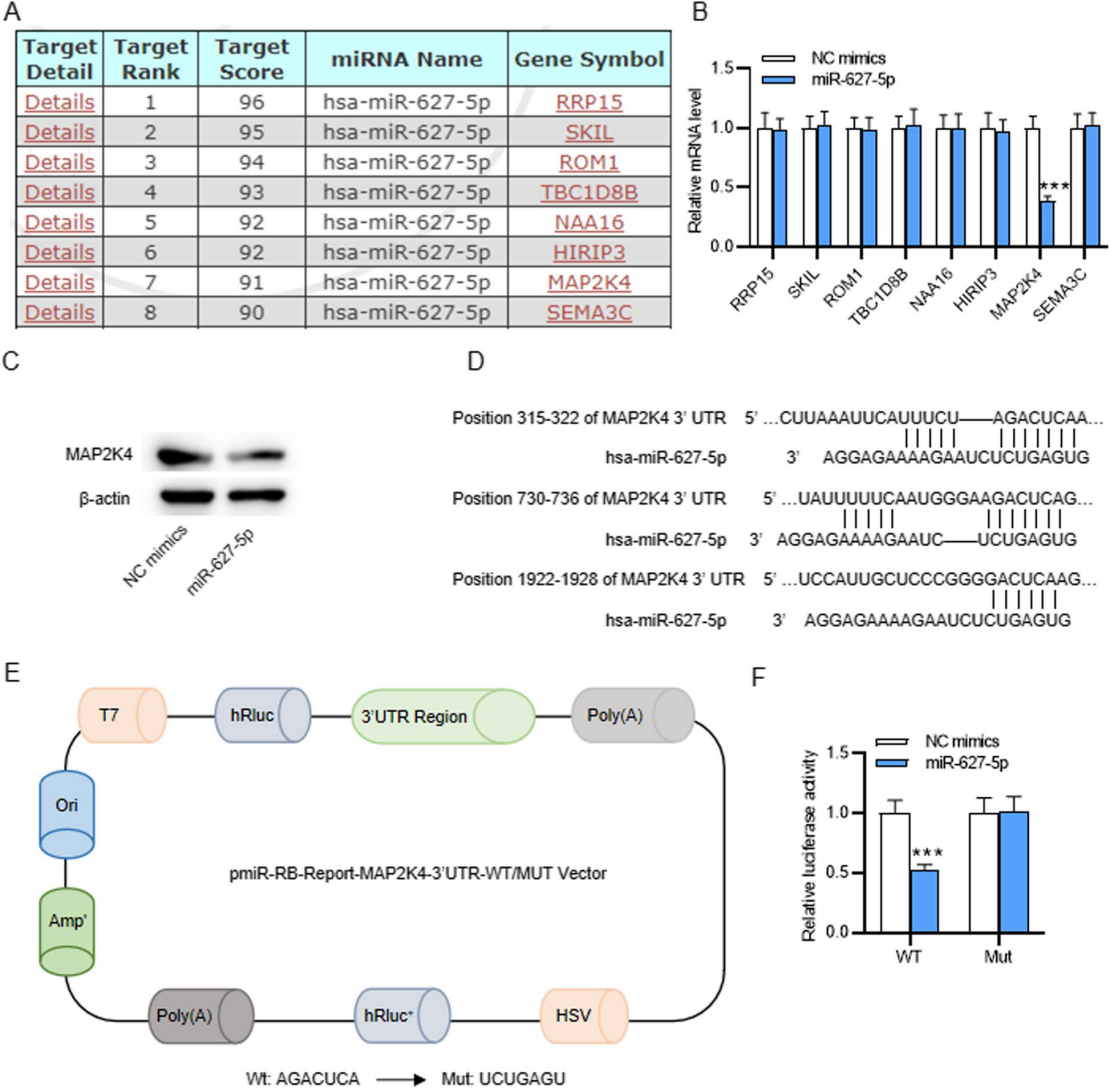
MAP2K4 directly targets miR-627-5p. **A** The downstream targets of miR-627-5p were predicted using miRDB database and the top eight were chosen for following qRT-PCR analysis. **B** qRT-PCR analysis of candidate gene expression in PASMCs after miR-627-5p overexpression. **C** Western blotting analysis of MAP2K4 protein level in PASMCs after overexpressing miR-627-5p. **D** The binding site of miR-627-5p on MAP2K4 3’UTR was predicted at TargetScan website. **E-F** The luciferase activities of pmiR-RB-REPORT-MAP2K4–3′UTR-Wt/Mut vectors in PASMCs after miR-627-5p overexpression were assessed through luciferase reporter assay. ***p<0.001

To further understand the regulatory mechanism of miR-627-5p on PASMC proliferation and migration, downstream targets of miR-627-5p was predicted using the miRDB database (http://mirdb.org/), and the top eight were selected (Fig. 3A). PCR analysis revealed that overexpressing miR-627-5p in PASMCs notably downregulated the level of MAP2K4, but failed to exert significant effect on the level of other seven mRNAs (Fig. 3B). Therefore, MAP2K4 was chosen for subsequent experiments. Through western blotting, overexpressing miR-627-5p was also discovered to cause a reduction in the protein level of MAP2K4 (Fig. 3C). Then, to verify the binding of miR-627-5p on MAP2K4 3’UTR predicted at TargetScan website (Fig. 3D), wild-type or mutant 3′UTR of MAP2K4 containing the miR-627-5p binding site were cloned into pmiR-RB-REPORT luciferase reporter vector for recombinant vectors pmiR-RB-REPORT-MAP2K4–3′UTR-Wt/Mut (Fig. 3E). MiR-627-5p overexpression markedly alleviated the luciferase activity of vectors containing MAP2K4-Wt, but exerted no significant effect on that of vectors containing MAP2K4-Mut (Fig. 3F). In summary, miR-627-5p targets MAP2K4.

### MAP2K4 reverses the inhibition of miR-627-5p overexpression on CSE-treated PASMC proliferation and migration

**Fig. 4:**
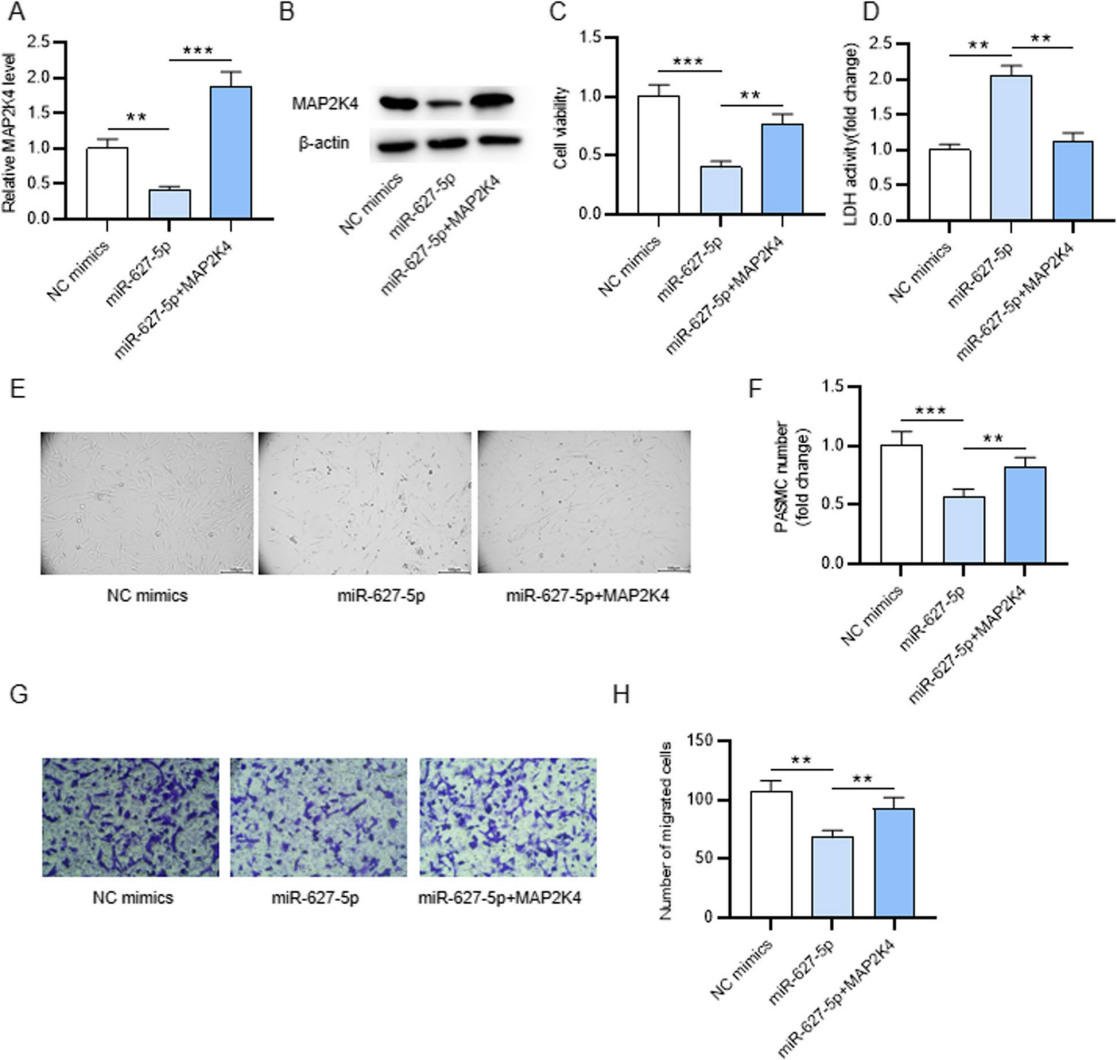
The role of the miR-627-5p/MAP2K4 axis in CSE-treated PASMC proliferation and migration. **A-B** qRT-PCR and western blotting analyses of MAP2K4 expression in PASMCs after indicated transfection. **C** CCK-8 assay of CSE-treated PASMCs viability after indicated transfection. **D** The examination of the lactate dehydrogenase (LDH) release of CSE-treated PASMCs after indicated transfection by using an LDH Cytotoxicity Assay Kit. **E-F** The images of CSE-treated PASMCs after indicated transfection were captured using a light microscopy and cell number was counted with a hemocytometer. **G-H** Transwell assay of CSE-treated PASMCs migration after indicated transfection. **p<0.01, ***p<0.001

Next, rescue experiments was performed to figure out whether miR-627-5p modulates CSE-treated PASMC proliferation and migration by regulating MAP2K4. We discovered that MAP2K4 mRNA and protein levels was downregulated after miR-627-5p mimics transfection, but was upregulated after miR-627-5p mimics and pcDNA3.1/MAP2K4 cotransfection (Fig. 4A-B). According to CCK-8 assay, MAP2K4 overexpression antagonized the suppression of miR-627-5p overexpression on the viability of CSE-treated PASMCs (Fig. 4C). Overexpressing miR-627-5p enhanced the LDH activity in CSE-treated PASMC culture supernatant, which was reversed after MAP2K4 overexpression (Fig. 4D). The images of PASMCs under the light microscopy showed that MAP2K4 overexpression antagonized the suppression of miR-627-5p overexpression on the number of PASMCs, suggesting that miR-627-5p repressed cell proliferation via downregulating MAP2K4 (Fig. 4E-F). Furthermore, the migrated number of PASMCs was reduced after miR-627-5p mimics transfection, but was elevated after miR-627-5p mimics and pcDNA3.1/MAP2K4 cotransfection (Fig. 4G-H). Overall, miR-627-5p suppresses CSE-treated PASMC proliferation and migration by downregulating MAP2K4.

### MiR-627-5p targets MAP2K4 to inhibit the PI3K/AKT pathway

**Fig. 5:**
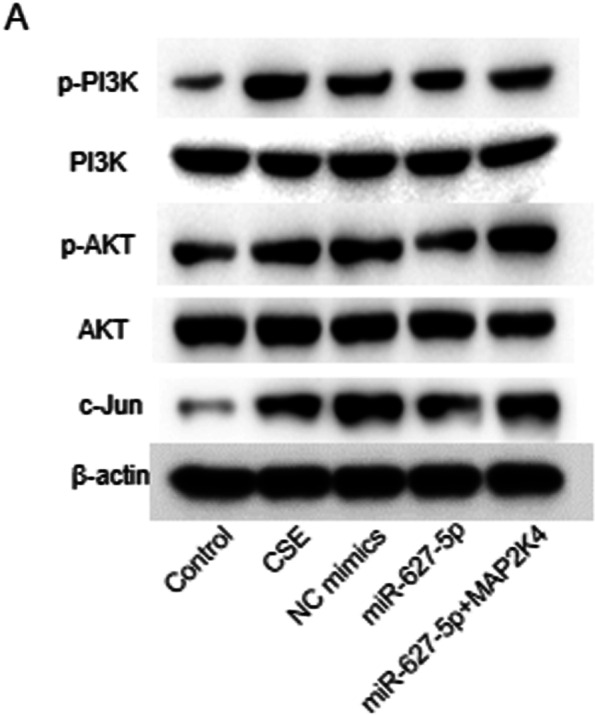
The regulation of the miR-627-5p/MAP2K4 axis on the PI3K/AKT pathway. **A** Levels of p-PI3K, PI3K, p-AKT, AKT and c-JUN proteins in 2% CSE-treated PASMCs after indicated transfection were assessed by western blotting

Finally, whether the miR-627-5p/MAP2K4 axis affects the PI3K/AKT pathway was evaluated. Western blotting was conducted for examining the levels of p-PI3K, p-AKT and c-JUN in CSE-treated PASMCs after different transfection. We first found that CSE treatment upregulated p-PI3K, p-AKT and c-JUN protein levels in PASMCs, showing that CSE activates the PI3K/AKT pathway. However, p-PI3K, p-AKT and c-JUN protein levels in CSE-treated PASMCs were downregulated after miR-627-5p mimics transfection, but was reversed after miR-627-5p mimics + pcDNA3.1/MAP2K4 cotransfection (Fig. 5A). This showed that MAP2K4 overexpression partially abolished the suppression of overexpressed miR-627-5p on the PI3K/AKT pathway. Therefore, miR-627-5p inhibit the PI3K/AKT signaling pathway via downregulating MAP2K4.

## Discussion

This study demonstrated the function of miR-627-5p/MAP2K4/PI3K/AKT regulatory axis in CSE-induced abnormal proliferation and migration of human PASMCs. An in vitro model of COPD was established by treating PASMCs with CSE [20], and we discovered that low concentrations (1 and 2%) of CSE facilitated PASMC viability, while high concentration (10%) of CSE repressed PASMC viability. Furthermore, the concentration that led to a peak in cell viability (2% CSE) also enhanced PASMC proliferation and migration. These findings are consistent with previous research findings [21, 22].

Till now, the investigation of miR-627-5p in human diseases has been limited, especially in pulmonary diseases. Previously, the role of miR-627 in pulmonary fibrosis was reported. MiR-627 attenuates TGFβ1-induced proliferation of normal human primary lung fibroblasts by downregulating HMGB1 and inhibiting the NF-κB pathway, thereby alleviating the development of pulmonary fibrosis [17, 23]. MiR-627-5p was reported to be downregulated in pulmonary artery homogenates isolated from lung tissues of COPD patients compared to non-smokers [16]. In this study, we similarly discovered that miR-627-5p expression was downregulated in CSE-stimulated PASMCs, the in vitro model of COPD. Through gain-of-function assays, overexpressing miR-627-5p reversed the promotion of CSE treatment on PASMC growth and migration, suggesting that miR-627-5p attenuates CSE-induced abnormal growth and migration of human PASMCs. Therefore, the alleviation of miR-627-5p against abnormal proliferation of PASMCs during the development of COPD is in line with that during the development of pulmonary fibrosis.

MAP2K4 is a member of the MAPK activator family [24]. The function of MAP2K4 in asthma, a respiratory disease caused by tracheal obstruction owing to aberrant growth and migration of airway smooth muscle cells (ASMCs), has been demonstrated [25, 26]. In asthma, downregulation of MAP2K4 facilitates the proliferative and invasive capabilities of ASMCs, while overexpression of MAP2K4 represses ASMC proliferation and invasion [27]. However, in our study, overexpressing MAP2K4 reversed the repression of miR-627-5p overexpression on PASMC proliferation and migration, which indicated that overexpression of MAP2K4 promotes the proliferative and migratory abilities of PASMCs. Therefore, the influence of MAP2K4 dysfunction on cell growth and migration are distinct in different cells.

The phosphatidylinositol-3-kinase/protein kinase B (PI3K/AKT) signaling pathway is one major pathway that modulates the proliferation of diverse types of cells [28, 29]. The PI3K/AKT pathway is associated with pulmonary vascular remodeling since it regulated the proliferation of several types of lung vascular cells including PASMCs [30–32]. MAP2K4 was reported to promote cell growth, migration, and invasion through activating the PI3K/AKT pathway and the downstream cell cycle-related protein, c-JUN [33]. In our study, overexpression of miR-627-5p suppressed the CSE-induced increase in the protein levels of p-PI3K, p-AKT and p-c-JUN, suggesting that miR-627-5p inhibited the CSE-induced activation of the PI3K/AKT pathway. However, MAP2K4 overexpression abolished the impacts of miR-627-5p on the protein levels of p-PI3K, p-AKT and p-c-JUN, suggesting that miR-627-5p attenuates abnormal growth and migration of human PASMCs by downregulating MAP2K4 and repressing the PI3K/AKT pathway.

Nevertheless, COPD is a symptom seen with long-term exposure to cigarette smoke, while the results of our study were based on a 24-h treatment with 2% CSE. This may be different from the actual pathogenesis of COPD. Therefore, the investigation of the role of miR-627-5p in the development of COPD in our study is only in preliminary phase. An animal model more similar to the pathogenesis of COPD requires to be established through the method of chronic exposure to cigarette smoke in future studies to further confirm the role of miR-627-5p in the progression of COPD.

## Conclusion

This study initially and innovatively confirmed the function of the miR-627-5p/MAP2K4/PI3K/AKT axis in COPD development. The finding showed that miR-627-5p restrains human PASMC dysfunction induced by CSE by downregulating MAP2K4 and repressing the PI3K/AKT pathway. Our study will provide a better understanding of miR-627-5p-mediated pathogenesis of COPD, which might provide novel insights for COPD treatment. Nevertheless, our study is still in preliminary phase and there also exist some limitations. First, the lack of in vivo experiments restricts the confidence of the findings in this study. Second, the investigation of upstream molecules regulating miR-627-5p might be focused on in future studies.

## Data Availability

We declare that the data that support the findings of this study are available from the corresponding author upon reasonable request.

## Abbreviations

COPD: Chronic obstructive pulmonary disease
PASMCs: pulmonary artery smooth muscle cells
CSE: CS extract
CS: cigarette smoke
UTR: untranslated region
FRS2: factor receptor substrate 2
VEGF: vascular endothelial growth factor
MAP2K4: mitogen-activated protein kinase kinase 4
qRT-PCR: Quantitative real-time polymerase chain reaction
CCK-8: Cell counting kit-8
LDH: lactate dehydrogenase
ASMCs: airway smooth muscle cells
PI3K/AKT: phosphatidylinositol-3-kinase/protein kinase B
